# Diabetes and Cancer: A Twisted Bond

**DOI:** 10.3389/or.2024.1354549

**Published:** 2024-05-21

**Authors:** Mihai Cosmin Stan, Doru Paul

**Affiliations:** ^1^ Emergency County Hospital Rm. Vâlcea, Râmnicu Vâlcea, Romania; ^2^ Medical Oncology Department, University of Medicine and Pharmacy of Craiova, Craiova, Romania; ^3^ Weill Cornell Medicine, New York, NY, United States

**Keywords:** diabetes, cancer, hyperglycemia, neuroinflammation, metformin, short-chain branched amino acids

## Abstract

This paper presents an overview of the interconnection between various factors related to both cancer and type 2 diabetes mellitus (T2DM). Hyperglycemia, hyperinsulinemia, chronic inflammation, and obesity are involved in the development and progression of both diseases but, strong evidence for a direct causal relationship between diabetes and cancer, is lacking. Several studies described a relationship between hyperglycemia and cancer at the cellular, tissular and organismic levels but at the same time recent Mendelian randomization studies proved a significant causal relationship only between hyperglycemia and breast cancer. On the other hand, the association between both hyperinsulinemia and obesity and several cancer types appears to be robust as demonstrated by Mendelian randomized studies. Metabolic alterations, including the Warburg effect and excessive glucose consumption by tumors, are discussed, highlighting the potential impact of dietary restrictions, such as fasting and low-carb diets, on tumor growth and inflammation. Recent data indicates that circulating branched-chain amino acids levels, may represent novel biomarkers that may contribute to both better diabetes control and early pancreatic cancer detection. Understanding the underlying mechanisms and shared risk factors between cancer and T2DM can provide valuable insights for cancer prevention, early detection, and management strategies.

## Introduction

Two of the most prevalent chronic pathologies—cancer and T2DM—appear to be interconnected and both have a serious effect on a patient’s health. There is an ongoing debate regarding the details of this connection, which is currently being investigated analyzing common genetic and epigenetic risk factors. Several genes have been identified that increase the susceptibility both to T2DM and cancer development (e.g., TCF7L2, CDKN2A/B, AKT2, PPARG, PTEN and HNF1B) but the evidence for a common etiology of both conditions is scarce [1]. An exception is the positive association between TCF7L2 alleles and higher risk of both T2DM and breast [2] and colorectal [3] cancer; and, also the association between type 2 diabetes predisposing alleles, and lower risk of prostate cancer [4].

There are several risk factors common to both conditions like hyperglycemia, which can lead to the production of advanced glycated end products (AGEs) and oxidative stress; hyperinsulinemia, which typically results from either impaired insulin function or insulin from extra sources; the inflammatory process; and obesity [5]. In T2DM insulin levels are high and may confer a higher cancer risk to these patients through its mitogenic effects, and, by the same token, anti-diabetic medications that lower insulin levels may be beneficial in cancer treatment [6]. Lack of physical activity and being overweight or obese are well-known risk factors for developing cardiovascular disease and diabetes [7], and at least thirteen types of cancers have been found to be related to obesity [8]. Diabetes is also closely associated with obesity and, obesity and tumorigenesis are related through a number of biological mechanisms some of them directly related to diabetes [9]. For example, increased levels of unbound IGF-1 protein are caused by obesity-related insulin resistance and hyperinsulinemia, which may also trigger the insulin and IGF-1 receptor signal transduction pathways, which may ultimately lead to tumor growth [10]. Although classically, insulin and IGF1 have been considered the major link between diabetes and cancer, as opposed to hyperglycemia [11], recently it has been demonstrated that high glucose levels increase the rate of cell mutation and decrease its capacity for repair [12]. As a result, the cell becomes more vulnerable to oxidative DNA damage and disruption of DNA integrity. Through a variety of mechanisms, including chemoresistance, drug deactivation, an impact on drug pharmacokinetics and dosages, and reduced immune responses, hyperglycemia may also diminish or even obstruct the effectiveness of cancer therapy. Moreover, compared to patients without hyperglycemia, cancer patients with high blood sugar have a greater proportion of metastatic disease and worse outcomes [13]. In a recent analysis of 37,993 patients from the Chinese National Health Interview Survey database, among all cancer survivors, patients with diabetes had a greater risk of all-cause mortality [homologous recombination (HR) 1.35, 95% CI = 1.27–1.43], cancer-specific mortality (HR: 1.14, 95% CI = 1.03–1.27), CVD mortality (HR: 1.36, 95% CI = 1.18–1.55), diabetes related mortality (HR: 17.18, 95% CI = 11.51–25.64), and kidney disease mortality (HR: 2.51, 95% CI = 1.65–3.82), compared with individuals without diabetes [14]. A meta-analysis of eight studies comprised of 4,342 patients, demonstrated that compared to patients without hyperglycemia, patients with elevated random blood glucose levels had lower disease-free survival (DFS) and overall survival (OS) [15].

## Pathophysiology of Cancer in Relation to Hyperglycemia

**FIGURE 1:**
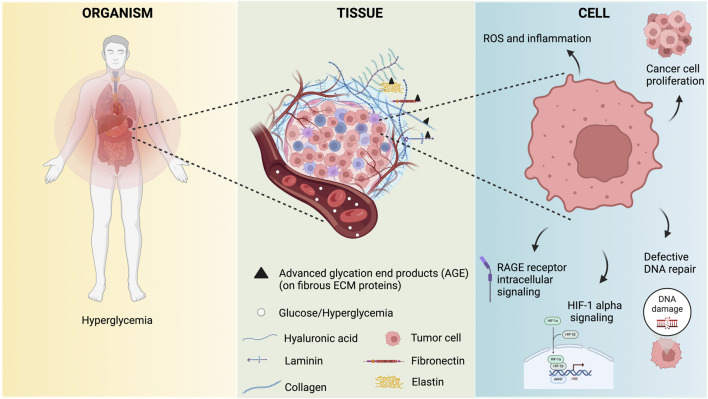
Organism levels impinges by hyperglycemia related to oncogenesis.

Cancer has been described as a wound that does not heal [16] and hyperglycemia is closely interfering with cellular repair mechanisms. Hyperglycemia impacts the organism at three levels related to oncogenesis: cellular, tissue, and systemic (Figure 1).

Analyzing the cellular level, several studies have shown that hyperglycemia accelerates cell multiplication and slows cell repair mechanisms, by modifying the cell’s sensibility to oxidative DNA damage and disruption of DNA integrity [17]. As a result, the major DNA repair pathways—base excision repair (BER), nucleotide excision repair (NER), mismatch repair (MMR), homologous recombination (HR), and non-homologous end joining (NHEJ)—are activated, enabling cells to repair DNA damage [12]. Hypoxia-inducible factor 1-alpha is modified by hyperglycemia, which stimulates a number of DNA repair-related genes, including the NER genes, and, as a result, genomic instability is enhanced in patients with type 2 DM [18]. Due to the interference of the error-prone NHEJ repair mechanism, these defects cause an accumulation of mutations, which is consistent with the detrimental effects of high glucose and the link between diabetes and cancer [12, 19]. Hyperglycemia also encourages the development of glycated moieties in different tissues, such as advanced glycation end products (AGE), which are the end result of a non-enzymatic reaction between reducing sugars and the amino groups of proteins, lipids, or nucleic acids. By reacting with DNA bases and producing ROS, NFkB, the AGE receptor (RAGE), or inflammation, AGE, and its precursors, can build up and cause DNA damage, which can then contribute to carcinogenesis and initiate pancreatic cancer and hepatocellular carcinoma (HCC) [13, 20]. TP53 induced apoptosis and double-strand DNA breaks are induced by excessive glucose metabolism in cells, possibly through oxidative stress and ROS production [21]. In normal colon cells, under folate-deficient conditions, high glucose increases the number of micronuclei, nucleoplasmic bridges, and nuclear buds, which contributes to genomic instability [22]. In addition to having a direct impact on genetic stability, hyperglycemia also results in epigenetic dysregulation, which sets off a series of downstream signaling cascades and raises the risk of neoplastic transformation [23]. Hyperglycemia also causes aberrant gene expression by altering the epigenome, leading to aggressive tumor progression that persists even after glycemic control is therapeutically achieved [24].

High blood sugar levels cause also **tissue-level changes** that alter the extracellular matrix. These changes are incompletely understood, and links to stop tissue induced carcinogenesis are being sought [25]. Glycation, a non-enzymatic interaction between sugars and the amino groups of proteins, lipids, and nucleic acids, results in advanced glycation end products (AGEs) [26]. By altering enzymatic activity, disrupting conformation, and interfering with the ligand-receptor interaction, glycation can impair normal protein function [27, 28]. These changes at the protein level can affect cell signaling and possibly influence tumor growth. Hyperglycemia-induced glycation has been demonstrated to cross-link and stiffen collagen matrix *in vitro* in addition to altering cell signaling [25]. Recent research has revealed a novel mechanism by which diabetes promotes the progression of breast tumors through glycation, and it suggests that one way to slow tumor growth in diabetics is by glycation targeting. AGEs are known to be inhibited by metformin, a first-line treatment for type II diabetes [29]. Compared to patients with type 2DM taking non-metformin antidiabetic regimens, patients on metformin have a roughly one-third lower cancer incidence and mortality rate [30].

Hyperglycemia has also a significant influence at the **organism level.** For example, several studies demonstrated the hyperglycemia impact on the immune system. Hyperglycemia is related to cytokine production suppression, phagocytosis decrease, immune cells dysfunctions, and failure to eradicate microorganisms [31]. Several studies have demonstrated that unbalanced type 2DM is the cause of inadequate cytokine production, such as IL-2, IL-6, and IL-10, which is crucial for both the body’s defense against pathogens and its capacity to adjust its immune response [32]. Additionally, it was discovered that obese leptin receptor-deficient mice and high-fat diet (HFD)-induced hyperglycemic mice produced less IL-22 than normal mice [33]. IL-22 is involved in innate host defense and tissue-protective and regenerative functions and, as a consequence, these mice are prone to infections. It has been also shown that diabetic hyperglycemia reduces the ability of macrophages and other leukocytes to destroy pathogens by significantly increasing endogenous production of tumor necrosis factor (TNF-α) and IL-6 [34]. It was also shown that the proportion of toll-like receptor (TLR-2), which is acting in *pathogen recognition* was smaller in diabetic mice [35], and normal in patients with controlled glycemia [31]. A large number of studies outlined how hyperglycemia may lead to neutrophil dysfunction, including defects in ROS production, impairment of neutrophil degranulation, inhibition of immunoglobulin-mediated opsonization, reduced phagocytosis, and defects in NET formation [31]. Hyperglycemia is also known for macrophage dysfunction [35], natural killer cell dysfunction [36], and inhibition of antibodies and complement effectors [37], which causes diabetic patients to become immune compromised, making them less able to handle all infection challenges [31].

## Cancer Influencing Diabetes and Viceversa

A century ago Warburg demonstrated for the first time that even in the presence of excess oxygen, several cancer cell types use glycolysis instead of oxidative phosphorylation [38]. In a different study, the researchers concluded that high consumption of calories made some cancers more aggressive, while diet made tumor aggression lower [11]. John Claras recently proposed in a provocative paper that tumor growth may be stimulated as an adaptive mechanism for consuming excessively circulating glucose, and once the metabolic event has been overcome, tumor growth is inhibited [39]. It is interesting to note that recent research indicates also that dietary restrictions, such as fasting and low-carb diets, do seem to slow tumor growth and reduce tumor size, suggesting that these lifestyle decisions can be used to lessen chronic inflammation and the related oncogenic signaling from the microenvironment to cancer [11]. If hyperglycemia contributes to tumor growth in some types of cancer [39], hypoglycemic-inducing methods like exercise may also reduce the risk of developing cancer [41].

The connection between diabetes and pancreatic cancer is well known for decades and is considered both a risk factor and an early sign of the disease [42]. Which one comes first? The two conditions are closely related and once one of them is present the risk of the other is increased. Apoptosis of beta cells caused by pancreatic stellate cells is a risk factor for pancreatic cancer via the islet fibrosis it induces [43, 44]. Reversely, the release of adrenomedullin, a possible mediator of beta cell dysfunction, is increased in pancreatic cancer and may lead to early diabetes [45]. According to some evidence, resection of pancreatic cancer may improve pre-existing diabetes in some cases of new-onset diabetes but not in patients with long-standing diabetes [46]. This finding appears to be uniquely related to pancreatic cancer as resection of pancreas in chronic pancreatitis does not improve pre-existing diabetes [47].

Another important aspect is the relationship between the metastatic process and T2DM. The vascular endothelium, whose function is disturbed in T2DM, plays an important role in the metastatic cascade [48]. In T2DM an increase in the permeability of the blood vessels may occur through an increase in advanced glycation end products and vascular inflammation [49]. Also, hyperglycemia leads to an increase in the Von Willebrand factor in the vascular endothelium promoting tumor cell adhesion and transendothelial tumor cell movement and the development of metastases [50]. A Japanese study suggested that diabetes mellitus may be associated with liver metastasis of colorectal cancer through the production of a biglycan-rich cancer stroma [51], negatively affecting the prognosis, representing another intriguing theory about the relationship between CRC and diabetes [52].

The role of inflammation in both type T2DM and cancer has generated increasing interest in targeting inflammation to improve prevention and control of these diseases [53, 54].

The Finnish Diabetes Risk Score (FINDRISC) is a functional tool designed in Finland to identify people at high risk of developing T2DM. An article published recently found that a higher FINDRISC is related to increased cancer incidence and mortality, a risk factor that is partially mediated by low-grade inflammation [55].

Recent research has shown that hyperglycemia may promote perineural invasion (PNI) in a number of malignancies, especially pancreatic carcinoma. Neuroinflammation is a well-known diabetic complication that causes nerve damage [56]. Neuronal glucose levels can increase by up to 4-fold in diabetic hyperglycemia. Intracellular glucose may damage neurons if spikes of hyperglycemia occur [57, 58]. In a hyperglycemic setting, levels of oxidative stress and proinflammatory substances may lead to nerve injury and an inflammatory reaction, simultaneously promoting cancer cell proliferation, migration, and metastasis. A similar pathophysiologic process has been described in diabetic women with ovarian cancer [59]. For this reason, as an example, recommendations are currently in place to use lower doses of Paclitaxel, a chemotherapeutic agent known to provoke or worsen neuropathies [60].

## Epidemiology

Pancreatic, colorectal, breast, endometrial, ovarian, hepatocarcinoma, and prostate cancer are only a few of the cancers that have been linked to diabetes and are significantly tied with obesity and insulin resistance [14, 61, 62].

On the other hand, several epidemiological studies have shown that certain cancers and T2DM are closely related and diabetes raises a person’s risk of developing cancer of pancreatic, liver, colon, breast, and endometrial cancer [14, 61].

The third most often diagnosed malignancy, colorectal cancer (CRC), accounts for more than 6% of all cancer cases worldwide [63]. There are several theories regarding the association between colorectal cancer and diabetes. In a large cohort study conducted in Canada between 2007 and 2015 on 44,178 participants with CRC, diabetes had a greater impact on non-cancer than cancer mortality risk for patients with CRC [64]. A large study published in 2016 in the British Journal of Cancer reported that diabetes mellitus is significantly associated with larger pancreatic tumors and also may elevate the overall risk of death of pancreatic cancer patients (HR of 1.19) [42]. Another epidemiological study found that sugar consumption is strongly correlated with an increase in both incidence and mortality of breast and colon cancer, independent of obesity [65]. Preclinical studies suggest that high-sucrose or high-fructose diets activate several pathways, including inflammation, glucose, and lipid metabolic pathways [66].

Interestingly, some cancers, such as those of the brain, buccal cavity, esophagus, lung, breast, urinary bladder, and larynx, demonstrated a null or decreased occurrence risk in diabetic patients in some studies [67]. It is noteworthy that several American and European studies have shown that individuals with type 2 diabetes have a lower risk of developing prostate cancer [68, 69]. Furthermore, patients with more than 10 years of T2DM duration showed a stronger protective effect [70]. Men with diabetes had lower testosterone levels [70] than men without the disease, and research has shown that testosterone is linked to a higher risk of prostate cancer [71]. Also, large studies found no correlation between T2DM and the risk of dying from cancers of the lung, bladder, stomach, cervix, esophagus, or leukemia [72, 73].

According to a five-country study on cancers in T1DM patients, there is a correlation between T1DM and the risk of multiple common cancers. Comparing non-sex-specific cancers to the general population, the estimated homologous recombination (HR) and 95% confidence intervals (CIs) for overall cancer were 1.15 (1.11, 1.19) for men and 1.17 (1.13, 1.22) for women [74].

Cancer incidence of liver, pancreas, kidney, esophagus, stomach, lung, thyroid, squamous cell carcinoma, and leukaemia significantly increased for both sexes with T1DM [75]. Incidence of non-Hodgkin’s lymphoma and colon cancer significantly increased for men [74]; while incidence of the ovary, esophagus, endometrium, vulva and vagina, and thyroid cancer significantly increased for women [76].

Likewise, when compared to the general population, men with T1DM had significantly lower incidences of testis and prostate cancer [74]. Melanoma, Hodgkin’s lymphoma, and breast cancer were notably less common in women with type 1 diabetes [74, 77]. Additionally, compared to the general population, patients with T1DM had an overall higher standardized mortality ratio for cancers, according to prior cohort studies [78].

Patients with T1DM did not exhibit a statistically significant increase in all-cause cancer mortality when compared to the general population, according to cohort studies conducted in the UK [75, 79] and the United States [80]. Even so, there was evidence of variation in the risk of some cancers depending on the nation and the length of T1DM [74].

Numerous illnesses affecting the exocrine pancreas are the cause of type 3c diabetes (T3cDM), also known as pancreoprivic diabetes [81]. Pancreatic cancer, acute and chronic pancreatitis, cystic fibrosis, trauma or pancreatectomy, fibrocalculous pancreatopathy, hemochromatosis, idiopathic forms, and uncommon genetic disorders are among the many causes of T3cDM. According to research by Pendharkar et al., about 0.11% of people with exocrine pancreas disorders had diabetes [82]. About 9.2% of diabetes patients were found to have T3cDM, according to Ewald N et al [83].

A thorough meta-analysis revealed a negative correlation between the length of diabetes and the relative risk of pancreatic cancer, with patients with a history of diabetes lasting less than a year having the highest risk of developing the disease [84]. It suggests that diabetes could have been the consequence of pancreatic cancer that went undetected [85]. In fact, up to 30% of pancreatic cancer patients have T3cDM [86]. Conversely, individuals with T3cDM as a result of pancreatic cancer may experience improved hyperglycemia if their cancer is successfully treated [46]. Furthermore, patients with chronic pancreatitis—the most common cause of T3cDM—have a 10- to 20-fold increased risk of pancreatic cancer; in patients with both chronic pancreatitis and diabetes mellitus, this risk is increased 33-fold.

T3cDM resulting from pancreatic cancer appears to be connected to the chemicals that cancer releases [87]. One of the main mediators of beta-cell toxicity in a pancreatic cancer cell-line study was found to be adrenomedullin [88].

Additionally, compared to the general population, patients with diabetes caused by pancreatic cancer had higher levels of adrenomedullin, according to a clinical study [45]. Furthermore, the upregulation of S100A8/A9 and connexin in pancreatic tissues may also attenuate the utilization of glucose [89, 90]. Additionally, it has been observed that pancreatic cancer patients with diabetes have high levels of interleukin-1β and tumor necrosis factor (TNF)-α in the tumor microenvironment [91], which may help to explain the compromised beta-cell function seen in these patients [92].

Mendelian randomization (MR) is an epidemiological technique that uses genetic variants to distinguish correlation from causation in observational data. MR became increasing popular in recent years and the direct association between diabetes and other factors associated with diabetes and cancer has been scrutinized in several studies using this methodology.

The MR studies demonstrate that the relationship between hyperglycemia, diabetes, hyperinsulinemia, inflammation, obesity and cancer is not straightforward. The authors of a large Japanese study using MR concluded that there is no strong evidence supporting a direct association between diabetes and the risks of total cancer, colon cancer, pancreatic cancer or liver cancer [93]. There seems to be a strong and very strong relationship between hyperinsulimia or obesity and cancer and a much weaker relationship between diabetes or hyperglycemia and cancer [1].

## Treatment and Prevention

Recent data indicate that metformin [94], besides its benefit for diabetic patients may have also a benefit in cancer patients. Metformin promotes the liver kinase B1 (LKB1)/AMPK signaling pathways and inhibits the mTOR pathway, it decreases insulin levels, protein translation, and circulating levels of insulin and IGF-1 in peripheral blood and may ameliorate dyslipidemia [95, 96]. Currently, the use of metformin in cancer prevention is still under scrutiny [95]. Large epidemiologic data suggest that metformin decreases the incidence of prostate, pancreas, liver, colon, thyroid, endometrial and esophageal cancers [97]. It may also improve the progression free survival of patients with ovarian cancer [39], the prognostic of patients with breast cancer [97] and the overall survival of patients with metastatic non-small cell lung cancer [98] and nasopharyngeal cancer [99]. Intriguingly it has been recently shown both *in vitro* and *in vivo* that Metformin may enhance the efficacy of check point inhibitors in lung cancer tumors harboring STK11 mutations [100]. The authors of a systematic review and meta-analysis reported significantly reduction in both overall cancer incidence and mortality in patients taking metformin [48]. Metformin’s potential to upregulate AMP kinase (AMPK), which inhibits mTOR and impairs angiogenesis as well as cell growth and proliferation—both essential for the progression of cancer—may explain how cancer growth is restricted but more mechanisms may be present, and sometimes it’s effect is counter-intuitive [101, 102]. While it was initially thought that AMPK might be a connecting link between diabetes and cancer, emerging studies indicate that the impact of metformin on cancer suppression, despite its activation of AMPK in cancer cells, is not definitive. This ambiguity is highlighted by the fact that metformin, through inhibition of complex 1, can increase glycolysis (Warburg effect), potentially promoting tumor growth in mice via elevating lactate and VEGF levels, although *in vitro* it leads to growth arrest because of enhanced extracellular acidification as a result of increased glycolysis [103]. Additionally, the role of AMPK in cancer is itself context dependent and appears contradictory [104], with some studies indicating its significant involvement in worsening cancer cell survival and promoting tumorigenesis [102, 105].

The number of preventable cancer deaths in patients with type two diabetes may be decreased by improving current screening programs for cancer or conducting more thorough examinations for non-specific symptoms [106]. Also, many malignancies can be potentially prevented by reducing their risk factors, which include a reduction in dietary consumption of sugars and carbohydrates [11], increased physical activity [41], and smoking cessation [42]. A recent large multinational cohort study demonstrated that both diabetes and cancer can be prevented through life-style interventions [107].

## Future Developments

Leucine, isoleucine, and valine, collectively known as branched-chain amino acids BCAA, are essential amino acids, both for the host and the tumor cells. It has been shown that elevated circulating BCAA levels are related to a number of conditions marked by insulin resistance (IR) and inflammatory response, including obesity and diabetes, both of which are known risk factors for cancer development [101]. Several large studies conducted in US and Japan have demonstrated that elevated circulating BCAA concentrations are early predictors for pancreatic ductal adenocarcinoma (PDAC) [108–110]. Recently, it has been also shown that metformin and sulphonylurea treatment results in lower BCAA levels [111]. Importantly, BCAA levels decreased as HbA1c levels improved, indicating that improper glucose metabolism may contribute to elevated BCAA levels. As a result, serum BCAA levels could be also a new indicator for assessing metabolic disorders and glycemic management [112].

The relationship between diabetes and cancer, where certain aspects remain under-addressed due to the current limitations in research. For instance, the potential dual role of anti-diabetic drugs as anti-cancer agents, as indicated by some articles and epidemiological studies, needs further exploration. The dual sword role of metformin, on one hand, known to activate AMPK – which in turn inhibits mTOR and suppresses proliferation—but, on the other hand, has also the potential to increase cancer progression, illustrates the intricacy of this relationship.

## Discussion

Cancer and T2DM are major public health concerns, and their association has gained significant attention in recent years. Although a direct causal relationship between the two conditions has not been proven, emerging evidence suggests shared risk factors and reciprocal indirect influence. Understanding these connections is crucial for developing preventive strategies and optimizing treatment approaches for both conditions. Hyperglycemia, hyperinsulinemia, chronic inflammation, and obesity contribute to both T2DM and cancer, influencing tumor growth, progression, and metastasis. BCAA levels may represent novel biomarkers that may contribute to both better diabetes control and early pancreatic cancer detection. Further research is needed to elucidate the complex relationship and explore the potential of lifestyle interventions and anti-diabetic medications in cancer prevention and management.
